# A Soluble ProDOT-Based Polymer and Its Electrochromic Device with Yellow-to-Green Color Switching Towards Camouflage Application

**DOI:** 10.3390/molecules29235585

**Published:** 2024-11-26

**Authors:** Shizhao Wang, Tao Yang, Haichang Fu, Yujie Dong, Weijun Li, Cheng Zhang

**Affiliations:** 1 College of Chemical Engineering, Zhejiang University of Technology, Hangzhou 310014, China; wangsz@zjut.edu.cn (S.W.); m15853364983@163.com (T.Y.); dongyujie@zjut.edu.cn (Y.D.); 2 Collaborative Innovation Center of Yangtze River Delta Region Green Pharmaceuticals, Zhejiang University of Technology, Hangzhou 310014, China; 3 Taizhou Biomedical and Chemistry Industry Institute, Taizhou 318000, China; hcfu@tzc.edu.cn

**Keywords:** ProDOT, conjugated polymer, electrochomic camouflage, electrochromic device

## Abstract

Yellow-to-green electrochromic color switching plays a key role in the intelligent adaptive camouflage under the visible light environment in future military camouflage applications. Here, we designed and synthesized a soluble electrochromic conjugated pDPTD polymer, mainly based on perylo[1,12-bcd]thiophene and the novel ProDOT groups. The pDPTD polymer displayed a yellow-to-green electrochromism with large optical contrast and fast switching times. Based on the pDPTD polymer film, a yellow-to-green electrochromic device was achieved, showing an orange-yellow color at −0.4 V with L*a*b* color coordinates of 88.5, 18.5, and 34.2 and a pale green color at 0.7 V with L*a*b* color coordinates of 85.6, −4.8, and 11.5, together with a large optical contrast of 43.5% and fast switching times of 2.4/3.2 s. These results indicated that the pDPTD polymer could serve as a potential electrochromic material for yellow/green system camouflage applications.

## 1. Introduction

Electrochromic conjugated polymer materials have been widely investigated for their greatly promising potential applications in electronic display [1,2,3,4,5] areas such as electronic color paper [6,7], electronic skins [8], outdoor energy-saving advertisement boards, etc. [9,10,11]. Electrochromic materials, due to their excellent color-changing performance, are considered one of the primary solutions for achieving intelligent adaptive camouflage [12,13,14,15,16]. Adaptive camouflage typically refers to objects adjusting their colors to blend with the surrounding environment to achieve perfect concealment [17,18,19], and it has become one of the key technologies to enhance military survivability [20,21]. However, the requirements of reversible switching between green and yellow camouflage colors in the visible light range [22,23,24], as well as excellent cycling stability, fast switching speed, and outstanding flexibility, are essential in practical application [25,26]. Conjugated polymers, due to their unique advantages, are considered one of the most likely materials to be applied in military camouflage [27,28].

Current studies for EC conjugated polymer materials mainly focus on the exploration of new materials and devices with yellow/green electrochromism, a fast switching time, and high cyclic stability [29,30,31,32]. In 2009, Leclerc et al. [33] reported three solution-processable carbazole-based conjugated polymers capable of green-to-brown switching. Based on the synthesized polymers, adaptive camouflage electrochromic devices with different pixel sizes and colors were prepared. In 2016, Meng Hong’s research group [34] synthesized D-A-D-type conjugated polymers with reversible switching between grass green and sandy yellow using EDOT as the donor unit and quinoxaline as the acceptor unit, and horizontally color-changing chameleon fabric devices were further prepared. Li Yao et al. [35] deposited flexible WO_3_ films on Au/nylon 66 porous substrates. By achieving reversible color switching between transparent and blue WO_3_ film and overlaying with the yellow Au substrate, reversible yellow-green switching could be achieved when the device was subjected to 1.5 V and −1.5 V voltages. Our previous work [28,36] reported the yellow-green switchable dual-polymer electrochromic devices using yellow-to-transmissive as well as green-to-transmissive polymers for camouflage applications, which could simulate environments encountered in various natural settings like forests, wilderness, and deserts.

**Scheme 1:**
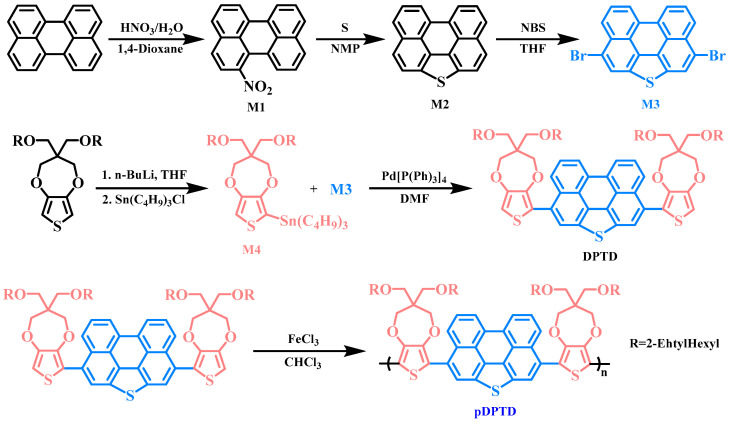
The molecular structures and synthesis route of branched monomer DPTD and pDPTD polymer.

Herein, we reported a soluble conjugated polymer, pDPTD, with yellow-to-green electrochromism based on ProDOT and perylo[1,12-bcd]thiophene, which was synthesized via FeCl_3_-catalyzed chemical polymerization (Scheme 1). Polymers that contain ProDOT can change from colored to colorless normally. The inclusion of perylo[1,12-bcd]thiophene allows pDPTD polymers to transition from yellow to green for camouflage, together with a large optical contrast and fast switching time. Using pDPTD polymer film as an electrochromic layer, the yellow-to-green electrochromic device was also achieved. The experimental data and analysis will be shown in detail as follows.

## 2. Results and Discussion

### 2.1. Polymer Synthesis and Film Preparation

**Figure 1:**
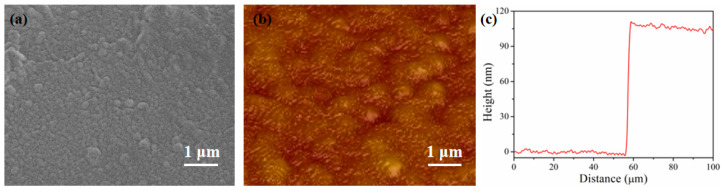
(**a**) FE-SEM image, (**b**) AFM image, and (**c**) step profiler analysis for pDPTD film.

The molecular structure of the electrochromic pDPTD polymer was mainly composed of ProDOT and perylo[1,12-bcd]thiophene groups, in which a relatively large twist angle between the two groups was constructed in order to obtain a high energy gap for yellow color. The synthesis route of the polymer can be found in Scheme 1. The pDPTD polymer was synthesized mainly by the coupling reaction between the DPTD monomer under the catalysis of FeCl_3_, which was obtained by the coupling reaction between self-synthesized bromo-substituted perylo[1,12-bcd]thiophene and the organotin compound of the ProDOT group. All the intermediate and terminal compounds were fully characterized, and the molecular weight of the pDPTD polymer was measured to be 27.7 kDa (Mn) using the GPC method. Using CHCl_3_ as a solvent, polymer material pDPTD could be soluble with high concentrations, which could enable the solution processing of polymer materials for the fabrication of film and devices through the spin-coating method, as shown in Figure 1. The concentration of the pDPTD polymer solution in the spin coating is 15 mg/mL in chloroform with 800 rpm/min. The finally obtained polymer film was estimated to be 110 nm in thickness using the step profiler.

### 2.2. Electrochemical Properties

**Figure 2:**
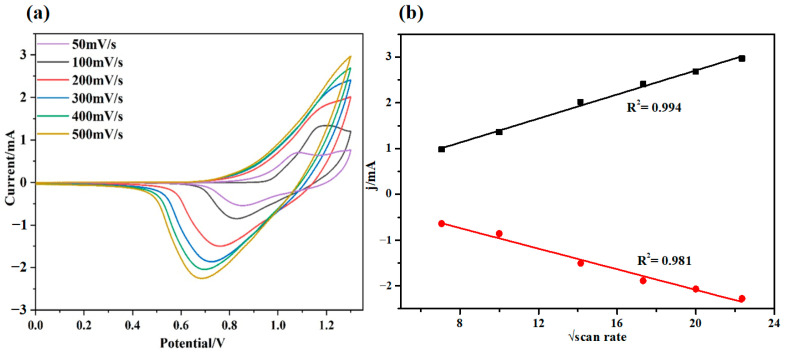
(**a**) CV curves of pDPTD at different scan rates from 50 to 500 mV/s in the 0.1 M TBAPF_6_/ACN solution. (**b**) The relationship between the peak current density and the square root of the scan rate.

The electrochemical properties of pDPTD polymer film were measured by carrying out the cyclic voltammetry experiment in acetylene containing 0.1 M TPAPF_6_ in the three-electrode system. The pDPTD polymer displayed a typical couple of reversible redox peaks around 1.1 V/0.8 V, as shown in Figure 2, and it could be seen that the pDPTD polymer film showed good oxidation and reduction activity. From the electrochemical CV curves, the onset oxidation potential of the pDPTD polymer film was estimated to be around 0.85 V. The relatively higher redox potential of the pDPTD polymer film might be ascribed to the introduction of perylo[1,12-bcd]thiophene groups because such a large planar conjugated group could obviously introduce a relatively larger twisted angle between adjacent perylo[1,12-bcd]thiophene and ProDOT groups owing to the steric hindrance and thus decrease the conjugated degree of the whole main chain of the polymer structure. Another unavoidable aspect was the intrinsic high oxidation potential of individual perylo[1,12-bcd]thiophene groups.

Figure 2 exhibited the CV curves of polymer film at different scan rates between 50 mV/s and 500 mV/s in an acetonitrile solution containing 0.1 M TPAPF_6_. When the scanning rates of the electrochemical CV test were increased from 50 mV/s to 500 mV/s, the electrochemical redox peak of the polymer film increased gradually in current intensity, but at the same time, the redox peak tended to be asymmetrical. As shown in Figure 2, through the relationship between the scanning speed and the peak current intensity, it could be seen that there was a good linear relationship between the peak current density and the square root of the scan rate of the polymer film. These data showed that the electrochemical process of the pDPTD polymer film was reversible and was controlled by diffusion throughout the process.

### 2.3. Electrochromic Properties

**Figure 3:**
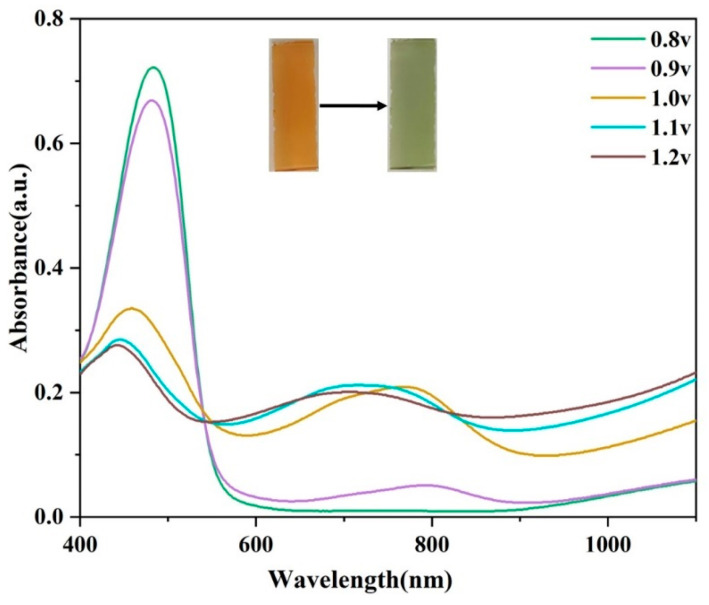
UV-vis spectroscopies of pDPTD at varying applied potentials in a 0.1 M TBAPF_6_/ACN solution (insets are the pictures of the color change in two polymer films under different potentials).

The UV spectra of the prepared polymer film pDPTD were studied as shown in Figure 3. The pDPTD polymer film displayed a broad absorption spectrum with λ_max_ at 484 nm, which should be attributed to the π-π absorption of the polymer main chain based on the weak conjugation between the ProDOT and perylo[1,12-bcd]thiophene groups. According to the absorption spectra, an optical band gap of the pDPTD polymer could be estimated to be 2.2 eV. The polymer film showed an obvious yellow color, which was consistent with the absorption spectra and high optical energy gap. The yellow color belongs to the three primary colors of the subtractive system (CMY) and should be used in electronic paper displays and yellow-green color camouflage applications.

The electrochromic properties of pDPTD films were measured by carrying out the spectroelectrochemical experiment in a 0.1 M TBAFP_6_/ACN solution. As shown in Figure 3, as the applied potential of pDPTD film increased, the original peak around 484 nm in UV absorption spectra decreased gradually in intensity, and a new peak around 700 nm appeared. When the potential was stable at 1.2 V, two obvious absorption peaks around 400/700 nm and one absorption spectra valley around 530 nm in the whole visible-light spectra range (400–800 nm) were finally observed, and the pDPTD film showed a green color. Thus, an electrochromism occurred in the pDPTD polymer films with a color change from the original yellow to green. Unlike those transparent oxidative states of ProDOT-based electrochromic polymers, the green color in the oxidative state of the pDPTD polymer was very rare. This was thought to be ascribed to the introduction of the perylo[1,12-bcd]thiophene group because it was not easily oxidized in itself, and the steric hindrance between it and the ProDOT groups could further decrease the conjugation of the polymer main chain in an oxidative state. An obvious fact could be concluded that the yellow-to-green electrochromism of the pDPTD polymer would make it of great potential in electrochromic camouflage applications.

**Figure 4:**
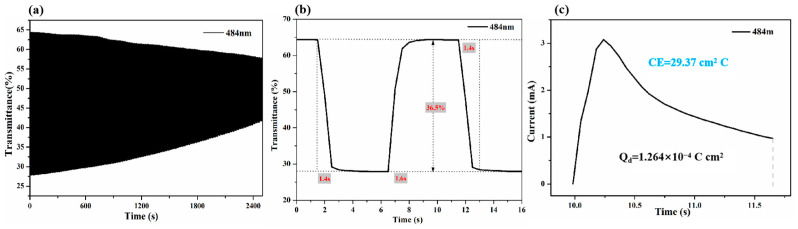
(**a**) The electrochromic stability for 250 cycles, (**b**) switching times of pDPTD under potentials between 0 V and 1.2 V (95% of the full switch), and (**c**) coloration efficiencies of pDPTD.

Kinetic studies were carried out to further elucidate the electrochromic properties of optical contrast and switching times for the pDPTD polymer under a repeated stepping potential between 0 V and 1.2 V with a residence of 5 s in an acetonitrile solution containing TBAFP_6_. As shown in Figure 4a, the optical contrast was ~36.5% for the pDPTD polymer monitored at the maximum absorption wavelength (484 nm). In addition, the long-term electrochromic cyclic stability of pDPTD film was also investigated by prolonging the stepping time with the stepping potential between 0 V and 1.2 V, as shown in Figure 4. The polymer films reveal the gradually decreased optical contrast as the stepping cycles increased, and finally, the optical contrast (at 484 nm) of pDPTD film decreased to ~15% after 250 cycles, which retained 42% of the original optical contrast level. The poor cycling stability of pDPTD films could be attributed to their high oxidation potential and the imbalance in redox charge injection and removal. This resulted in the breakage of the main polymer chain, leading to a loss of redox activity and a significant decrease in contrast. The switching time was defined as the time required to reach 95% of the full switch of the transmittance, and the coloring and discoloring time of the pDPTD polymer film were calculated to be ~1.6 s and ~1.4 s, respectively (Figure 4b). The large optical contrast and fast switching time were benefits of its military camouflage application. In addition, pDPTD can be energy saving in applications with a coloration efficiency of 29.37 cm^2^ C^−1^ (Figure 4c).

Coloration efficiency is calculated by Formula (1) as follows [37]:

(1) $$CE(\eta )=\frac{∆OD}{Q_{d}}=\frac{lg(\frac{T_{OX}}{T_{neut}})}{Q_{d}}$$

where ΔOD is the optical density, Q_d_ is the injected/ejected charge per area during a redox step, and T_OX_ and T_neut_ are the percent transmittance in the neutral and oxidized states, respectively.

The properties of yellow-green switchable electrochromic materials prepared by different methods are summarized in Table 1. Comparative analysis with the existing literature reveals that pDPTP exhibits superior optical contrast and a shorter switching time. These results underscore the exceptional suitability of pDPTP as an electrochromic material for military camouflage applications, suggesting that perylo[1,12-bcd]thiophene is a versatile building block for innovative electrochromic material design.

### 2.4. Electrochromic Device and Application

**Figure 5:**
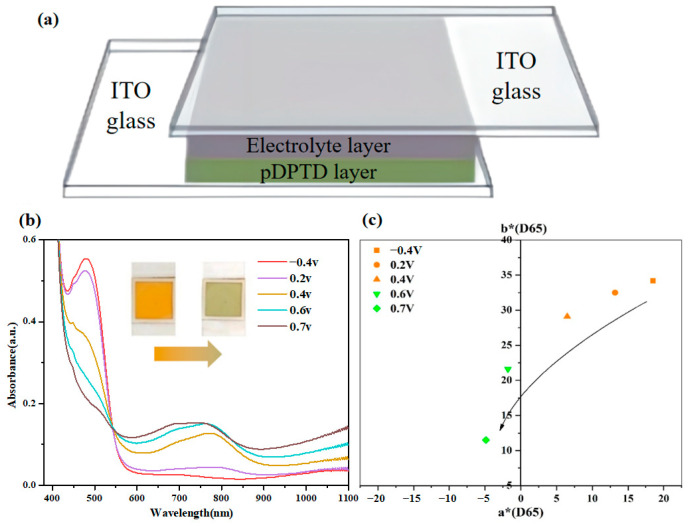
(**a**) The structure of yellow-to-green ECDs, (**b**) UV-vis spectroscopies (inset: the digital photos of the ECDs at different applied voltages), (**c**) CIE L*a*b* color coordinates of the ECDs (size: 2.5 × 2.5 cm^2^) from −0.4 V to +0.7 V.

Considering the yellow-to-green electrochromism of pDPTD polymer material, the double-layer device structure was adopted to achieve the yellow-to-green switched electrochromic device in order to avoid the color confusion of the ion storage layer. For the electrolyte layer, a typical gel electrolyte containing polymethylmethacrylate (PMMA), propene carbonate (PC), 0.05 M FeCl_3_, and 0.1 M TBAFP_6_ was used. The pDPTD polymer material and gel electrolyte layers were sealed between two ITO glasses to obtain the final electrochromic device, as shown in Figure 5.

The spectral and chromaticity data of the device were tested using a combination of an electrochemical workstation and a UV–visible spectrophotometer or a colorimeter, where different voltages were applied using the electrochemical workstation, and the absorption spectra or chromaticity of the device at different voltages were measured using the UV–visible spectrophotometer or colorimeter. As shown in Figure 5, the device exhibited an obvious absorption peak around 480 nm at −0.4 V, and when the applied potential increased from −0.4 V to 0.7 V, the absorption peak at 480 nm decreased in intensity, and a new peak around 700 nm appeared and increased in intensity. Thus, the device showed a very similar spectra character to those of pDPTD polymer material. At −0.4 V, the device showed an orange-yellow color, with L*a*b* color coordinates of 88.5, 18.5, and 34.2. At 0.7 V, the device displays a pale green color, with L*a*b* color coordinates of 85.6, −4.8, and 11.5. During the whole electrochromism process, the device’s color changed gradually from yellow to green, as shown in Figure 5, indicating that the device could show several diverse yellow and green colors.

**Figure 6:**
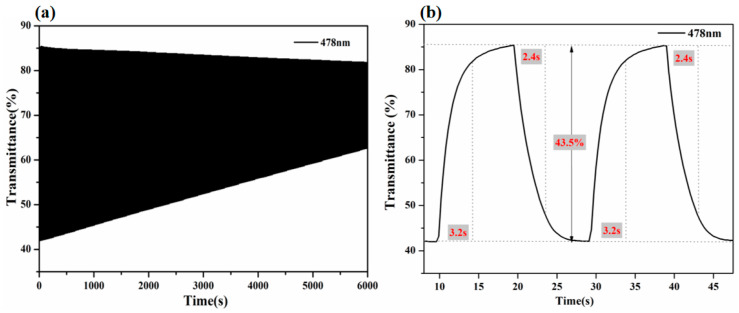
(**a**) The electrochromic stability for 300 cycles and (**b**) switching response and optical contrast tests of ECDs (size: 2.5 × 2.5 cm^2^) between −0.4 V and +0.7 V.

The cyclic stability, optical contrast, and switching times of the device were also tested using the combination of an electrochemical workstation and a UV–visible spectrophotometer. As shown in Figure 6, the device exhibited a large optical contrast of 43.5% at 478 nm, and the coloring and discoloring times of the device were calculated to be 3.2 s and 2.4 s, respectively. In comparison to the pure pDPTD polymer material, the device showed a similar color change with larger optical contrast and close switching times. Additionally, when the device underwent 300 cycles under the applied voltages between −0.4 V and 0.7 V, its optical contrast decreased from 43.5% to 20.1%, retaining 46% of its original optical contrast level, which was much better than that of pDPTD polymer material. The device exhibited good stability due to its packaging and the proper alignment of various functional layers within.

**Figure 7:**
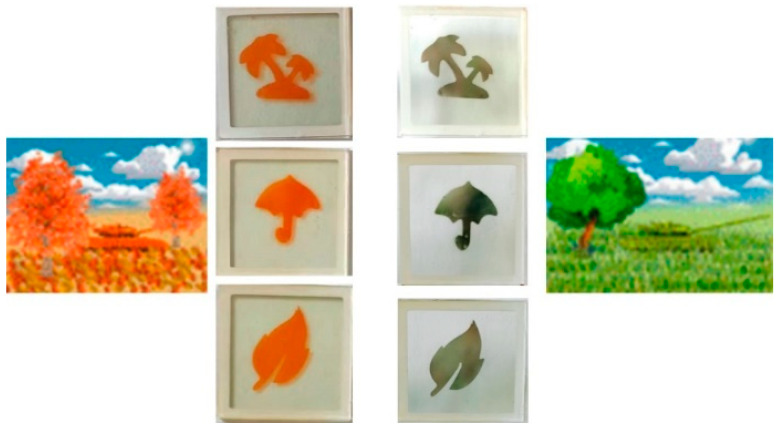
Camouflage effects of ECDs under the applied potential of −0.4 V and 0.7 V.

In order to fulfill the demand for military camouflage, patterned electrochromic devices were also tried to be prepared. The pDPTD polymer material exhibited good solubility in chloroform, allowing for the preparation of patterned electrochromic film through spray coating. Here, patterned films such as coconut trees, small umbrellas, and leaves were successfully obtained based on pDPTD polymer material, as shown in Figure 7. Then, these patterned films were further used to achieve the patterned electrochromic devices. They could all transform between yellow and green under the applied potential of −0.4 V and 0.7 V, respectively. When in the yellow-color state, these devices could simulate autumn trees and fallen leaves. While in the green-color state, they could simulate spring trees and growing leaves. Based on this characteristic, such electrochromic devices could be potentially applied in military camouflage areas, and the pDPTD polymer material should serve as the yellow/green system electrochromic camouflage materials.

## 3. Materials and Methods

### 3.1. Chemicals

All the reagents or chemicals were commercial products without further purification. Indium tin oxide (ITO) glass substrates (Liaoning Huite Technology, Yingkou, China, RS ≤ 10 Ω^−1^, area: 0.9 cm × 4 cm or 2.5 cm × 4.0 cm) were cleaned by ultrasonication in a series of solvents, including 0.5% aqueous sodium hydroxide, distilled water, ethanol, toluene, and acetone solutions for 15 min, respectively.

### 3.2. Characterization

The 1H NMR (400 MHz or 500 MHz) spectra were investigated by an Ascend 400-MHz or Ascend 500-MHz instrument (Bruker, Zurich, Switzerland). The molecular weights and polydispersity indices (PDIs) of polymers were investigated by a Waters Alliance e2695 gel permeation chromatograph (GPC, Waters Corporation, Milford, MA, USA). Surface morphologies were recorded by a Field Emission Scanning Electron Microscope (Zeiss, Jena, Germany). The thickness of the film was measured by a step profiler (Bruker, Switzerland). Electrochemical properties of the corresponding films were performed on a CHI 660E electrochemical analyzer (Chenhua, Shanghai, China). The spectroelectrochemistry and kinetic test of electrochromic films were investigated by the Shimadzu UV-1800 spectrophotometer (Shimadzu, Kyoto, Japan) integrated with the CHI 660E electrochemical analyzer. Switching times and cyclic stability tests of materials involved the following. In a conventional three-electrode cell, a voltage of 0 V is applied to the film for 5 s; then, an oxidation voltage is applied for 5 s, and the cycle is repeated. The CM-3600d spectrophotometer (Konica Minolta, Tokyo, Japan) was used to represent the color coordinates in the CIE 1976 L*a*b* Color Space. All electrochemical tests were performed in a conventional three-electrode cell using a polymer-coated indium tin oxide (ITO) electrode as the working electrode, a platinum wire as the counter electrode, and an Ag/AgCl in saturated KCl solution as the reference electrode.

### 3.3. Synthesis

The electrochromic polymers were synthesized via the reaction route, as shown in Scheme 1. Their detailed experimental procedures were shown as follows.

#### 3.3.1. Synthesis of Compound **M1**

Perylene (5.00 g, 19.80 mmol) and 1,4-dioxide (200 mL) were added into the dry 500 mL bottle and heated up to 50 °C; then, fuming nitric acid (4.50 mL) and water (2.0 mL) were added, and the mixture was stirred for 0.5 h. After the reaction was cooled to room temperature, 1000 mL of water was added, and the mixture was extracted with dichloromethane. The product was then dried with Na_2_SO_4_ and purified on a silica gel column (petroleum ether CH_2_Cl_2_ 1:1 as eluent) to obtain the final product as a brown-red crystalline (1.13 g, yield 19%, Figure S1). ^1^H NMR (DMSO, 400 MHz) δ 8.56 (d, *J* = 7.5 Hz, 1H), 8.53 (d, *J* = 7.6 Hz, 1H), 8.04–7.96 (m, 4H), 7.84–7.73 (m, 3H), 7.69 (t, *J* = 7.8 Hz, 1H), 7.58 (t, *J* = 7.9 Hz, 1H). MS (EI), and m/z: 297.3 [M]^+^. 

#### 3.3.2. Synthesis of Compound **M2**

Sulfur (1.72 g, 53.84 mmol) and *N*-methylpidopirazone (80 mL) were mixed, and compound 1 (1.60 g, 5.36 mmol) was added under a nitrogen atmosphere. The mixture was heated to 180 °C and stirred for 3 h. After, the reaction was cooled to room temperature, filtered, and washed three times with hexane (3 × 200 mL) to obtain the final product as a bright yellow powder (1.06 g, yield 70%, Figure S2). ^1^H NMR (DMSO, 400 MHz) δ 8.81 (d, *J* = 7.6 Hz, 2H), 8.34 (d, *J* = 8.8 Hz, 2H), 8.22 (d, *J* = 8.0 Hz, 2H), 8.10 (d, *J* = 8.8 Hz, 2H), 7.92 (t, *J* = 7.8 Hz, 2H). MS (EI), and m/z: 282.4 [M]^+^.

#### 3.3.3. Synthesis of Compound **M3**

NBS (1.26 g, 7.08 mmol) was added in batches into the mixture of compound 2 (1.00 g, 3.54 mmol) and tetrahydrofuran (20 mL) and stirred for 5 h under the dark condition. After the reaction was completed, the mixture was washed with water and obtained as the final product as a bright yellow powder (1.13 g, yield 90%). Compound M3 was very poorly soluble, and its NMR data could not be obtained in common deuterium reagents; thus, it was used directly for the next reaction without further purification.

#### 3.3.4. Synthesis of Compound **M4**

The n-butyllithium solution (8.91 mmol, 2.4 mol/L) in hexane solvent was dropwise added into the mixture of compound 5 (3.19 g, 7.24 mmol) and dry THF (30 mL) under a nitrogen atmosphere at −78 °C. After stirring for 30 min, tributyl tin chloride (2.92 g, 9.00 mmol) was added and further stirred for 2 h. The mixture was then further stirred over night at room temperature and run simply in an aluminum oxide column (CH_2_Cl_2_ as eluent) to obtain the raw product as a pale yellow oil for the next reaction without further purification.

#### 3.3.5. Synthesis of Compound DPTD

A mixture of compound 3 (0.50 g, 1.13 mmol), compound 6 (1.60 g, 3.39 mmol), and Pd (PPh_3_)_4_ (35 mg) in 30 mL of dry DMF was heated with reflux for 15 h under a nitrogen atmosphere. Then, the mixture was extracted with dichloromethane, dried with Na_2_SO_4_, and further purified on a silica gel column (petroleum ether CH_2_Cl_2_ 3:1 as eluent) to obtain the final product as an orange-red oil (1.04 g, yield 80%, Figure S3). ^1^H NMR (500 MHz, CDCl_3_) δ 8.67 (d, *J* = 7.6 Hz, 1H), 8.19 (d, *J* = 8.4 Hz, 1H), 8.15 (s, 1H), 7.86 (t, *J* = 8.0 Hz, 1H), 6.65 (s, 1H), 4.17 (s, 3H), 4.10 (s, 2H), 3.51 (dd, *J* = 9.4, 3.0 Hz, 7H), 3.28 (d, *J* =5.7 Hz, 7H). MS (APCI), and m/z: 1159.7 [M]^+^.

#### 3.3.6. Synthesis of the pDPTD Polymer

Compound **7** (0.5 g, 0.43 mmol) was added into the chloroform solution (20 mL) containing FeCl_3_ (0.64 g, 3.95 mmol) and stirred for 24 h at 30 °C. Then, the appropriate amount of hydrazine hydrate was added, and the mixture was poured into the methanol solvent. After filtering, the solid was purified by methanol, acetone, hexane, and chloroform solvents in order using the Soxhlet extractor. At last, the final chloroform solution was evaporated to obtain the polymer product (Figures S4 and S5). GPC data are Mn = 27.7 kDa, Mw = 96.2 kDa, and PDI = 3.47.

## 4. Conclusions

A soluble electrochromic pDPTD polymer was successfully synthesized based on the ProDOT and perylo[1,12-bcd]thiophene groups. The electrochemistry, photophysical, and electrochromic properties were also fully studied. The pDPTD polymer displayed a yellow-to-green electrochromism with a large optical contrast and a fast switching time. Using pDPTD polymer film as an electrochromic layer, a yellow-to-green electrochromic device was achieved, showing an orange-yellow color at −0.4 V with L*a*b* color coordinates of 88.5, 18.5, and 34.2 and a pale green color at 0.7 V with L*a*b* color coordinates of 85.6, −4.8, and 11.5, together with a large optical contrast of 43.5% and fast switching times of 2.4/3.2 s. These results indicated that the pDPTD polymer could serve as a potential electrochromic material for yellow/green system camouflage applications.

## Figures and Tables

**Table 1:** Data on yellow-green materials in this work and other yellow-green EC materials.

	Working Voltage	Optical Contrast	Switching Times (s)	Cyclic Stability	Ref.
FTP	0~1 V vs. Ag/AgCl	18.54%	2.95/0.90	retain 80.66% (after 600 cycles)	[36]
4TBC	0~1.2 V vs. Ag/AgCl	32.00%	3.87/1.33	retain 67.80% (after 30 cycles)	[28]
pPHBT	0~1.4 V vs. Ag/AgCl	25.00%	4.2/1.5	retain 40.00% (after 500 cycles)	[38]
6Cz6F-PI	0.0~1.09 V vs. Ag/AgCl	31.00%	3.87/0.97	<85% (after 10 cycles)	[39]
PBDTC	0.0~1.4 V vs. Ag/AgCl	12.00%	2.29/1.20	-	[40]
pDPTP	0.0~1.2 V vs. Ag/AgCl	36.50%	1.6/1.4	retain 42.00% (after 250 cycles)	This Work

## Data Availability

Data will be made available upon request.

## Supplementary Materials

The following supporting information can be downloaded at https://www.mdpi.com/article/10.3390/molecules29235585/s1, Figure S1: ^1^H NMR spectra of M1; Figure S2: ^1^H NMR spectra of M2; Figure S3: ^1^H NMR spectra of DPTD; Figure S4: ^1^H NMR spectra of pDPTD; Figure S5: The molecular weight of the pDPTD polymer.
