# Supplementary material for: A Soluble ProDOT-Based Polymer and Its Electrochromic Device with Yellow-to-Green Color Switching Towards Camouflage Application
Source: Molecules. 2024 Nov 26;29(23):5585. doi: 10.3390/molecules29235585 (PMC11643929; doi:10.3390/molecules29235585)
Supplement: Supplementary file 1 [file molecules-29-05585-s001.zip › molecules-3243759-supplementary.pdf]

Supporting information

A Soluble ProDOT-based Polymers and Its Electrochromic Device with Yellow-to-Green Color Switching towards Camouflage Application

Shizhao Wang [1,2], Tao Yang [1], Haichang Fu [3], Yujie Dong [1], Weijun Li [1,*] and Cheng Zhang [1,*]

[1] College of Chemical Engineering, Zhejiang University of Technology, Hangzhou 310014, China; wangsz@zjut.edu.cn (S.W.); m15853364983@163.com (T.Y.); dongyujie@zjut.edu.cn (Y.D.)
[2] Collaborative Innovation Center of Yangtze River Delta Region Green Pharmaceuticals, Zhejiang University of Technology, Hangzhou 310014, China
[3] Taizhou Biomedical and Chemistry Industry Institute, Taizhou 318000, China; hcfu@tzc.edu.cn
* Correspondence: liwj@zjut.edu.cn (W.L.); czhang@zjut.edu.cn (C.Z.)

1. Spectra of the compounds

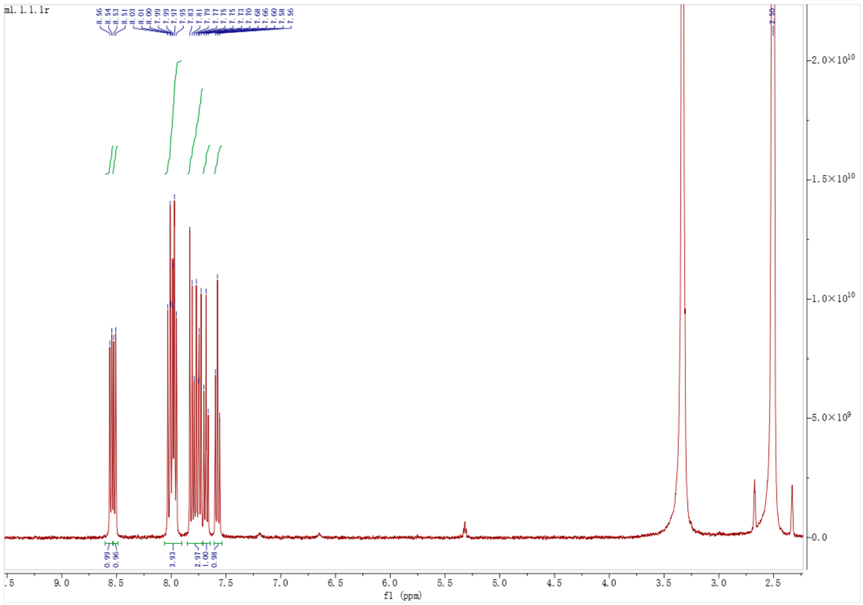

Figure S1. ^{1}H NMR spectra of M1 in DMSO.

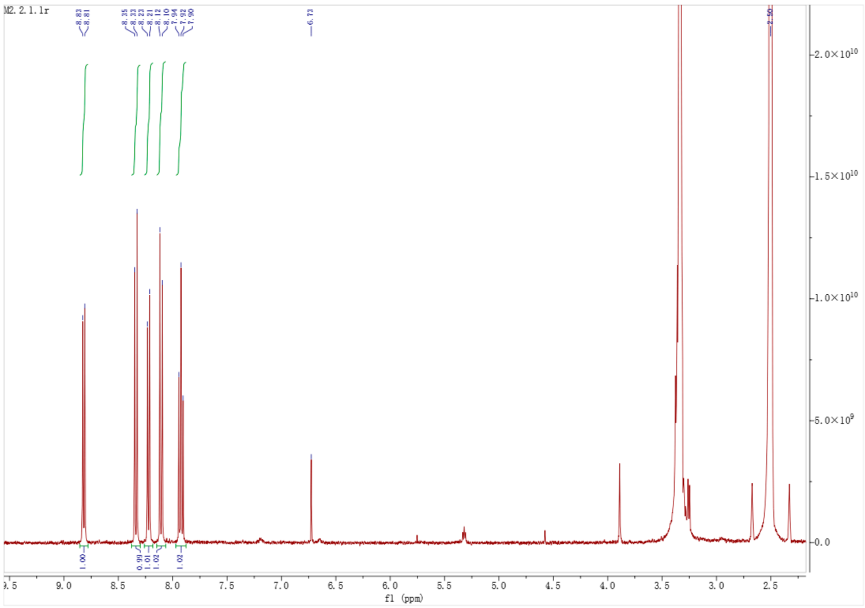

Figure S2. ^{1}H NMR spectra of M2 in DMSO.

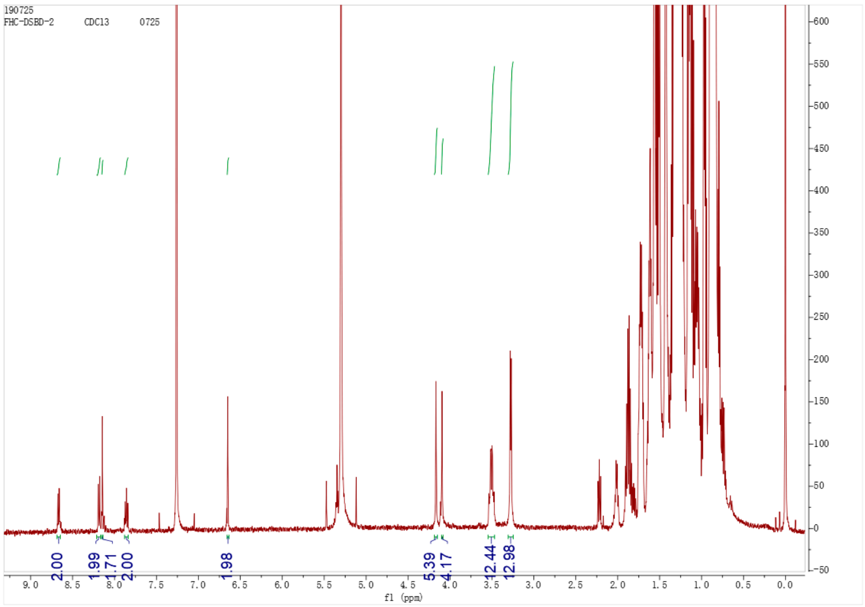

Figure S3. ^{1}H NMR spectra of DPTD in $CDCl_3$.

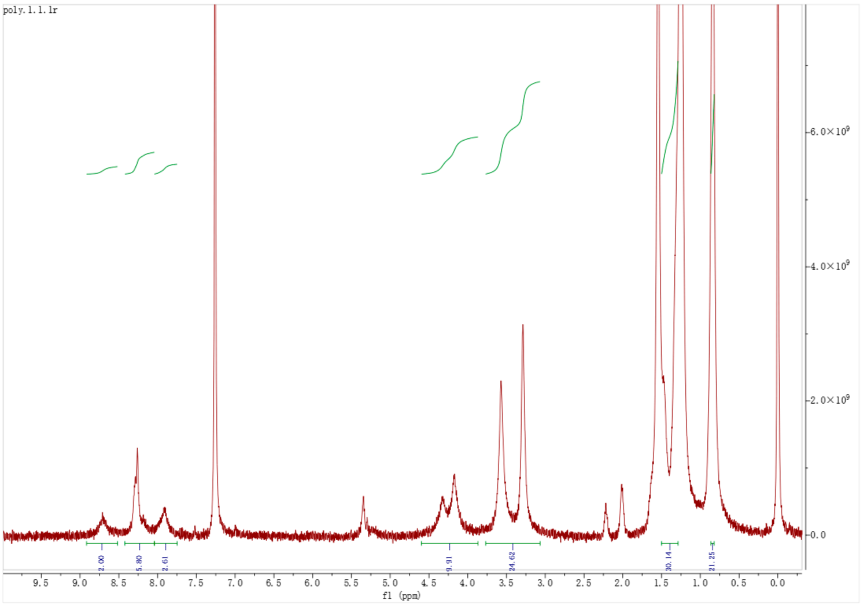

Figure S4. ^{1}H NMR spectra of pDPTD in $CDCl_3$.

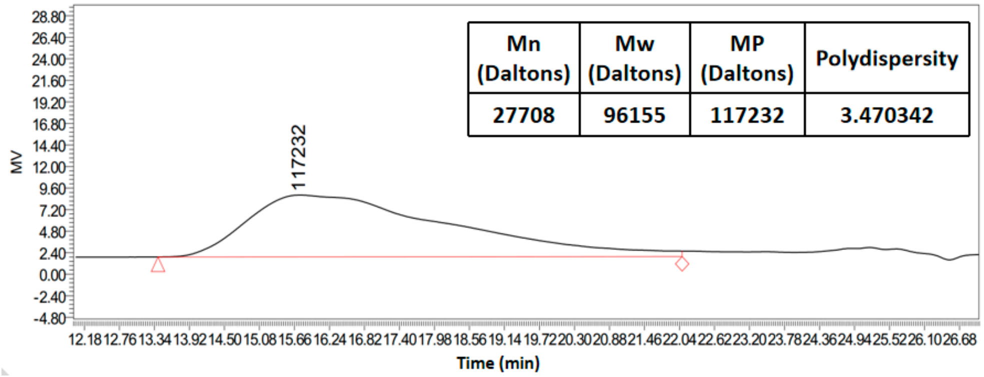

Figure S5. The molecular weight of polymer pDPTD by GPC method.

2. Preparation of the gel electrolyte

Take PMMA in the reagent bottle, then PC was added. The mixture was heated and expanded in the oven at 75℃ for 20 hours (record it as system A). Then the supporting electrolyte $TBAFP_6$ and $FeCl_3$ were added into the mixed solvent of acetonitrile (4 g) and dichloromethane (10 g), after ultrasonic for 5 hours until the electrolyte was completely dissolved (record it as system B). The system A and system B were mixed, and the resulting mixed solution was ultrasonic for 5h, and dried in a vacuum drying oven at 70℃ for about 3 hours to obtain the gel electrolyte.
